# Bilateral Transplantation of Allogenic Adult Human Bone Marrow-Derived Mesenchymal Stem Cells into the Subventricular Zone of Parkinson's Disease: A Pilot Clinical Study

**DOI:** 10.1155/2012/931902

**Published:** 2012-03-13

**Authors:** N. K. Venkataramana, Rakhi Pal, Shailesh A. V. Rao, Arun L. Naik, Majahar Jan, Rahul Nair, C. C. Sanjeev, Ravindra B. Kamble, D. P. Murthy, Krishna Chaitanya

**Affiliations:** ^1^Advanced Neuro Science Institute, BGS Global Hospital, Bangalore 560060, India; ^2^ANSA Research Foundation, Indiranagar, Bangalore 560038, India

## Abstract

The progress of PD and its related disorders cannot be prevented with the medications available. In this study, we recruited 8 PD and 4 PD plus patients between 5 to 15 years after diagnosis. All patients received BM-MSCs bilaterally into the SVZ and were followed up for 12 months. PD patients after therapy reported a mean improvement of 17.92% during “on” and 31.21% during “off” period on the UPDRS scoring system. None of the patients increased their medication during the follow-up period. Subjectively, the patients reported clarity in speech, reduction in tremors, rigidity, and freezing attacks. The results correlated with the duration of the disease. Those patients transplanted in the early stages of the disease (less than 5 years) showed more improvement and no further disease progression than the later stages (11–15 years). However, the PD plus patients did not show any change in their clinical status after stem cell transplantation. This study demonstrates the safety of adult allogenic human BM-MSCs transplanted into the SVZ of the brain and its efficacy in early-stage PD patients.

## 1. Introduction

Shaking Palsy (*Paralysis Agitans*) or Parkinson's disease (PD) was originally described by James Parkinson in 1817 as “Involuntary tremulous motion, with lessened muscular power, in parts not in action and even when supported; with a propensity to bend the trunk forwards, and to pass from a walking to a running pace: the senses and intellects being uninjured” [1].

Ever since the first description, scientists have pursued the causes and treatment of the disease. It is a chronic neurodegenerative disorder due to selective loss of dopaminergic neurons in the Substantia Nigra (SN) and the presence of proteinaceous inclusions known as Lewy bodies [2]. PD is recognized as one of the most common neurologic disorders, affecting approximately 15% of individuals older than 60 years. Between the ages of 75 and 84, that percentage may rise to almost 30%.

The causes of idiopathic Parkinson-disease (IPD) are believed to be a combination of genetic and environmental factors. Recent studies indicate that the pathogenesis includes a cascade of molecular and cellular events, oxidative stress, and release of reactive oxygen species (ROS), apoptosis, dysfunctioning of mitochondria and of the protein degrading system. Even immune-mediated mechanisms are being suggested for the progression and to explain the drug resistance that happens with time [2, 3].

The cardinal symptoms for PD are bradykinesia, rigidity, tremor, and instability which can be treated with dopamine replacement drugs. However, these drugs are unable to interrupt the progress of the disease and are ineffective against the disabling gait freezing, postural instability, lethargy, and lack of facial expressions. Also, over time there are drug-induced motor system complications; hence, it is suggested to delay the therapy till it significantly limits the patient's activities of daily living [4].

It is an established fact that much earlier to the clinical manifestations of the disease there are functional and structural changes in the nigrostriatal pathways which leads to a fall in the dopamine levels; releasing its inhibition and increasing the excitatory activities of the subthalamic nuclei and corticostriatal connections [1, 5]. Compensatory mechanisms are capable of maintaining the balanced neuronal output but not for long. Therefore, it was essential to search for alternate options to improve neuronal activity in the degenerated part of the brain. This lead to cell replacement therapies being identified as the most suitable option for PD as there is selective loss of dopaminergic neurons in the substantia nigra [6–10].

Over the years, numerous sources of dopamine-secreting cells like fetal mesencephalic tissue, human embryonic stem cells, and neural stem cells have been investigated with varying degrees of efficiency [6–8]. Although fetal mesencephalic tissue and embryonic stem cells showed a lot of promise, they are limited by the availability of fetal tissue and ethical concerns, persuading scientists to look deeper into the problem. This led to challenge the decade old hypothesis that regeneration is not possible in the brain. Stem cells were discovered in specific sites in the CNS such as the subventricular zone-SVZ (around the lateral ventricles) and their propensity to migrate to the traumatized areas of the nervous system. Numerous studies also confirm the differentiation capacity of stem cells into dopaminergic neurons in the presence of various external cues [11–13].

As a result, stem cells have emerged as a promising area helpful for tissue regeneration. Bone marrow mesenchymal stem cells (BM-MSCs) have the potential to differentiate into different lineages including functional dopaminergic neurons [14] without forming tumours. Animal studies illustrate that these cells have the property to migrate/home to the lesioned region as they respond to the chemoattractants released at the site [15–18].

An earlier report by our group in 2010 established the immediate and short-term safety of autologous bone-marrow derived mesenchymal stem cells in the transplantation therapy of PD and traumatic spinal cord injury patients [19]. Although the clinical improvement was only marginal, most of the patients experienced subjective well being without any notable side effects. Symptoms like freezing and facial expressions showed a tendency towards improvement. However, we noticed a variation in the BM-MSCs which were attributed to age and probably to long-standing disease. This encouraged us to carry out further studies in PD using BM-MSCs to overcome the variable in the previous study.

## 2. Materials and Methods

A clinical study was designed to determine the safety, feasibility, and efficacy of allogenic adult bone-marrow-derived mesenchymal stem cells in Parkinson's disease (PD) patients.

According to the national guidelines, Institutional Ethics Committee (IEC) approval was obtained for conducting the study. Necessary approvals for isolation, culturing, and transplantation of stem cells were also taken. Each patient who participated in the study was counseled on the procedure and informed consent obtained. The patient was screened for HIV, HBV, HCV, CMV, and VDRL by a nationally certified testing laboratory before being included in the study. All deviations to the protocol, drop outs, and adverse events were documented and informed to the IEC.

### 2.1. Study Design and Randomization

The study was conducted as a prospective, uncontrolled, one year, single centre safety, and efficacy clinical study of allogenic BM-MSCs bilaterally transplanted in patients diagnosed with PD.

### 2.2. Isolation of Mesenchymal Stem Cells from Healthy Adult Donors

#### 2.2.1. Selection of Healthy Donors

Healthy donors were selected according to the donor inclusion criteria and as per the guideline of International Society of Cell Therapy (ISCT). Healthy donors were either male or female in the age group of 18–30 years of age, able to understand the voluntary donation program, and ready to provide voluntary written informed consent. Donors were excluded if they have illness such as autoimmune disorders, tuberculosis, malaria and any other infection, any illness which precludes the use of general anesthesia, history of malignancy, diabetes, hypertension, significant heart disease, genetic or chromosomal disorders, history of any inherited disorders, hemoglobin less than 10, and pregnant women.

At the time of obtaining informed consent they were screened for infection with human immunodeficiency virus (HIV), hepatitis B (HBV), hepatitis C (HCV), cytomegalovirus (CMV), and syphilis (VDRL) using reverse transcriptase-polymerase chain reaction (RT-PCR) method and excluded if found positive. They were also tested for complete blood count (CBC), renal function test (RFT), liver function tests (LFT), blood glucose, chest X-ray, Echocardiogram, and Electrocardiogram (ECG).

#### 2.2.2. Isolation of Mesenchymal Stem Cells

BM-MSCs were isolated from healthy screened donors between the age of 18–30 years with informed consent. 60 milliliter of bone marrow was aspirated aseptically under local anesthesia from iliac crest of the healthy screened donors. The sample will be transported appropriately to the processing lab consisting of a class 1000 cGMP facility, and all sample processing was done in a class 100 biosafety cabinet. Bony spicules and particles were removed using a cell strainer and further diluted with DMEM-KO, centrifuged at 1800 rpm for 10 minutes at 20°C. The cells were resuspended with DMEM-KO and gently layered onto a density gradient solution (Lymphoprep, Axis Shield PoC AS, Oslo, Norway) to obtain an enriched mononuclear fraction. This was washed with DMEM-KO and centrifuged to collect the cells. The cells obtained were resuspended and plated in MSC complete culture medium consisting of Dulbecco's modified Eagle Medium (DMEM-KO) 10% fetal bovine serum (FBS) from preselected lots, and glutaMAX as described elsewhere [20]. The culture was maintained at 37°C in a humidified atmosphere containing 95% air and 5% CO_2_ and subcultured prior to confluency.

### 2.3. Subculturing and Expansion

Once 80% confluent these cells were dissociated with 0.25% trypsin/0.53 mM EDTA (Invitrogen) and further upscaled and expanded in order to provide the required number of cells to the patient. Briefly, trypsinized cells were reseeded at a density of 1000 cells per cm^2^ in cell stacks (Corning). After 14 days in culture, the cells reached 80% confluency and were ready for transplantation.

### 2.4. Quality Control Testing

MSCs were tested for quality control parameters such as Mycoplasma, Endotoxin, sterility and cell surface markers such as CD73, CD90, CD105, CD166, CD34, and CD45 markers using flow cytometry. They should be more than 80% positive for CD73, CD90, CD166, and CD105 but negative [<10%] for CD34 and CD45.

### 2.5. Characterization of Mesenchymal Stem Cells

#### 2.5.1. Immunophenotype

Immunophenotyping of the cultured BM MSC was performed using flow cytometry to identify the presence of specific cell-surface antigens. Briefly, BM MSCs were dissociated with 0.25% trypsin-EDTA and resuspended in wash buffer at a concentration of 1 × 10 cells/mL. Cell viability was measured by flow cytometry using 7-amino actinomycin D (7-AAD) 200 *μ*L cell suspension were incubated in the dark for 30 min at 4°C with saturating concentrations of phycoerythrin PE-conjugated antibodies. Appropriate isotype-matched controls were used to set the instrument parameters. After incubation, cells were washed three times with wash buffer and resuspended in 0.5 mL wash buffer for analysis. Flow cytometry was performed on a 5HT Guava instrument. Cells were identified by light scatter for 10000 gated events and analyzed. The following markers were analyzed: CD34-PE, CD45-PE, CD73-PE, CD105-PE, CD166-PE, and CD90-PE (BD Pharmingen, San Diego, CA, USA).

#### 2.5.2. Differentiation

The trilineage differentiation capacity of human BM MSC into osteoblasts, adipo cytes and chondrocytes was investigated to confirm mesenchymal properties. Briefly osteoblast differentiation was induced by culturing human BM MSC in Stempro Osteogenesis Differentiation kit (Life Technologies, USA) for 15 days as per the recommendations provided by the manufacturers. Fresh medium was replenished every 3 days. Calcium accumulation was assessed by Von Kossa staining. The differentiated cells were washed with DP BS and fixed with 10% formalin for 30 min. The fixed cells were incubated with 5% silver nitrate for 60 min under ultraviolet (UV) light and then treated with 2.5% sodium thiosulphate for 5 min. Images were captured using an Nikon Eclipse 80i microscope (Nikon Corporation, Towa Optics, New Delhi, India).

To induce adipogenic differentiation, human BM MSC were cultured for 21 days using Adipogenesis differentiation kit (Life Technologies, USA) as per the protocol recommended by the manufacturers. Medium was replenished every 3 days. Cells were fixed in 10% formalin for 20 min, and 200 *μ*L Oil Red O staining solution was added and incubated for 10 min at room temperature. The cells were rinsed five times with distilled water. The images were captured using a Nikon Eclipse 80i microscope (Nikon Corporation, Towa Optics, New Delhi, India). For chondrogenic differentiation, human BM MSC were cultured for 21 days using chondrogenesis differentiation kit (Life Technologies, USA) as per the manufacturer's recommendations and stained with Safranin O as specified. The images were captured using Nikon Eclipse 80i microscope (Nikon Corporation, Towa Optics, New Delhi, India).

#### 2.5.3. Karyotyping

To rule out any chromosomal aberrations during *in vitro* propagation of BM MSC, these cells were karyotyped prior to transplantation. The chromosomes were visualized using a standard G-banding procedure, and more than 200 cells were analyzed per sample and reported according to the International System for Human Cytogenetic Nomenclature (ISCN).

### 2.6. In Process Test

Prior to dispatching the cells for transplantation, a battery of in-process quality testing was performed on the cells. These include morphology, immunophenotyping cell surface marker analysis, endotoxin testing using LAL test, and mycoplasma using RT-PCR was also done. Only those cells fulfilling the ISCT criteria for MSCs were released for transplantation.

Any sample positive for endotoxin and mycoplasma was discarded immediately and appropriately.

### 2.7. End Product Test

The final cell suspension which was provided to the clinician for transplantation was again tested for cell surface marker analysis as mentioned above. In addition, karyotyping, endotoxin, and mycoplasma were also performed as mandatory quality testing. Cell viability was measured by flow cytometry using 7AAD (7-amino actinomycin D). Certificate of analysis (COA) was prepared, and cells were released along with documentation for transplantation.

### 2.8. Patient Selection

Subjects, both male and female between 18–80 years, were enrolled for this study. The patients were screened for HIV, HBV, HCV, CMV, and VDRL followed by inclusion criteria selection before participating in the trial. 8 PD patients and 4 PD plus syndrome patients were chosen for the trial (those patients diagnosed with multiple system atropy and progressive supranuclear palsy (PSP) have been classified under PD plus syndrome patients). This would help us understand the role of bone-marrow-derived mesenchymal stem cells in the early stages of the disease and in rapidly progressing PD plus syndrome patients.

Those patients who fulfilled the following inclusion and exclusion criteria were included for the study.

**Inclusion Criteria**

1. Should be in the age group of 18–80 years.
2. Should be fully conscious, alert, and oriented while providing consent.
3. Should show significant motor and nonmotor symptoms.
4. Subject should provide a written informed consent and agree to return for follow up.
5. Subject should be clinically diagnosed for Parkinson's disease and PD included disorders with motor complications despite adequate oral anti-Parkinsonian therapy.
6. Should be able to comply with and understand the required visit schedule.

**Exclusion Criteria**

1. Patient is suffering from Dementia (MMSE < 25).
2. The extent or severity of the disease is not measurable.
3. If the subject suffers form preexisting medical conditions such as bleeding disorders, and septicemia.
4. Patients with a past (within one year) or present history of psychiatric disorder.
5. If the subject has been enrolled in other investigational drug trial or has completed any trial within the last 3 months.
6. If hemoglobin < 10 gm/dL, serum creatinine < 2 mg/dL, serum total bilirubin < 2 mg/dL, and HbA1c < 7%.
7. Pregnant or nursing or women in child bearing age without adequate contraception.
8. The subject tested positive for HIV, HCV, HBV, CMV, or VDRL.

### 2.9. Clinical Evaluation

These patients were admitted 48 hours prior to the procedure, and a detailed clinical evaluation was performed including UPDRS, MMSE, gait and neuropsychological assessments. General physical examination and cardiac status of the patients were evaluated before inclusion into the study. MRI was done at the baseline and at 12 month follow up. MR Tractography was conducted at 3rd, 6th and 9th month follow up sessions.

### 2.10. MRI Imaging and Tractography

MRI imaging was done on a 1.5 5T (Wipro GE, Milwaukee, WI, USA) machine. Routine imaging was done with axial FLAIR and T2W images and Sagittal T1W images. DTI of brain was done in the axial plane using an 8-channel CTL array spine coil with the following parameters-25 directions EPI tensor imaging (TR 8500, TE: 97.6 *b* value: 1000 frequency: 128, phase 128, NEX-1, slice thickness: 5 mm with zero interslice gap and bandwidth: 250 kHZ).

### 2.11. Image Processing

Image processing was done using FuncTool software provided by GE and quantitative analysis was done to calculate fractional anisotropy using standard methods. ROI were placed in bilateral centrum semiovale, genu, splenium of corpus callosum, anterior limb of internal capsule, posterior limb of internal capsule, and cerebral peduncles (total of 12 ROI).

### 2.12. Statistical Analysis

Descriptive statistical analysis has been carried out in the present study. Results on continuous measurements are presented on Mean ± SD (min-max) and results on categorical measurements are presented in number (%). Significance is assessed at 5% level of significance. Student *t*-test (two tailed, dependent) has been used to find the significance of study parameters on continuous scale within each group.

### 2.13. Processing of Cells for Intracranial (IC) Transplantation

As mentioned in the earlier study [19] the cells were processed for transplantation. Briefly, after harvesting step, the total cell count was taken using a standard hemocytometer. The cells were washed several times with normal saline solution. Finally the cells were resuspended in saline containing 0.2% human serum albumin. The cell suspension (2 mL) was equally distributed into two 2 mL syringes and labeled. These were packaged in a sterile container and dispatched in a transportation container maintained at 22°C to the hospital for transplantation via the shortest route.

### 2.14. Surgical Procedure

The patient was positioned supine for the transplantation and the parts aseptically prepared. Under short propofol anesthesia bilateral frontal burr holes were drilled and small dural openings made. The sub ventricular zone was accessed through a standard brain cannula with CRW stereotactic frame or Stealth (Medtronic) navigation assistance. BM-MSCs, at a dose of 2 million cells/kg body weight, were transplanted into the brain and gelfoam placed over the dural defect prior to closing of the wound.

After operation the patients were observed in the neurointensive care unit for 24 hours following which they were shifted to the ward and discharged home on the 4th/5th day.

### 2.15. Evaluations and Follow-Up Schedule

The patients were followed up closely every three months for one year. They were assessed by an independent neurophysician and a movement disorders specialist. During each visit the patient was clinically examined, UPDRS score performed, neurologically assessed, and medications reviewed. At the final follow-up visit, that is, 12th month the patient would undergo a MRI scan to check for any structural changes in comparison to the baseline scan. The medication would be reviewed at each visit and adjusted based on the symptoms. Any adverse event would be reported to the concerned investigator and IEC.

## 3. Result

In this study, 12 patients were recruited according to the study design as per the inclusion and exclusion criteria mentioned above. This included 9 males and 3 females in the age group of 37–69 years. The duration of the disease varied between 3 and 15 years in the study group. Out of the 12 patients, 4 were diagnosed as PD plus and belonged to the older age group. The details of the patients who participated in this study are mentioned in Table 1.

2 million cells/kg body weight suspended in 2 mL of saline was implanted bilaterally into the subventricular zone using burr hole surgery technique. All patients tolerated the procedure well, there were no postoperative complications and were discharged within a week's time from the hospital. This indicates that there were no immediate cytotoxic effects due to implantation of allogenic bone marrow mesenchymal stem cells in to the sub-ventricular zone of the brain and the procedure was safe.

### 3.1. Allogenic BM-MSCs

3 adult healthy screened donors were recruited for the aspiration of bone marrow under general anesthesia. BM-MSCs were isolated and cultured as described in [20]. These cells were cryopreserved and appropriately propagated once the patients were recruited for the study. All the cells were assessed for their morphology, immunophenotype and differential potential.

### 3.2. Characterization of BM-MSCs

#### 3.2.1. Morphology and Immunophenotype

**Figure 1:**
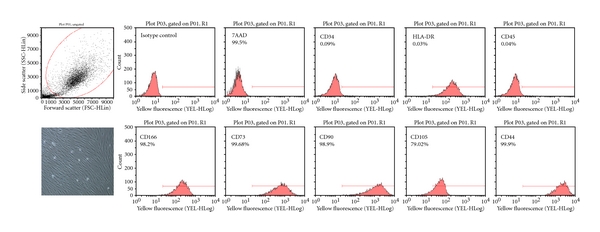
Characterization of adult BM-MSCs prior to transplantation as per ISCT criteria. It shows the plastic adhered spindle-shaped fibroblast like appearance of adult BM-MSCs in culture (lower left panel). And the surface expression of CD markers (top panel: negative markers and lower panel: positive markers).

The cells displayed a typical spindle shaped fibroblast-like appearance as shown in Figure 1 and flow cytometric analysis shows the cell surface expression of CD markers (as per ISCT) as shown in Figure 1. The cells were found to be CD34^−^/CD45^−^/CD73^+^/CD90^+^/CD105^+^/CD166^+^ as depicted in Figure 1.

#### 3.2.2. Multipotent Characteristics

**Figure 2:**
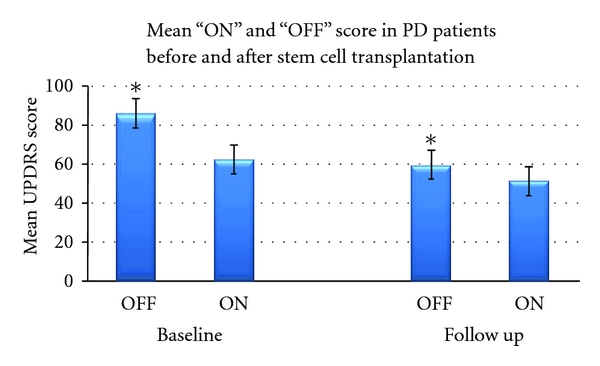
Shows the mean ± SD of the UPDRS score of the PD patients during “OFF” and “ON” periods at baseline and after stem cell transplantation. The scores have been assessed during the screening visit and final visit 12 months after transplantation. *Represents the level of significance.

In order to ensure that the BM-mesenchymal stem cells maintain their typical properties, the trilineage differentiation capacity of these cells was demonstrated. The cells were found to undergo adipogenic, osteogenic and chondrogenic differentiation as determined by Oil Red O stain, Von Kossa stain and Safranin O stains, respectively, (Supplementary data (Figure  2) will be available online at doi: 10.1155/2012/931902).

This set of analysis confirms that the cells being used for the clinical study are truly mesenchymal in nature.

#### 3.2.3. Karyotype

**Figure 3:**
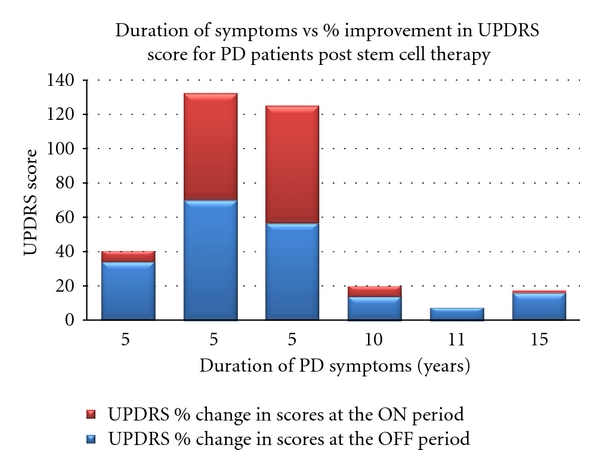
Illustrates the improvement in UPDRS scores for PD patients before and after stem cell transplantation. The graph highlights the fact that the patients treated early at the onset of the disease (5 years) have shown significantly better improvement which correlates clinically. The patients that were treated between 10–15 years after the diagnosis of PD did not show any significant improvement.

All the samples used for transplantation were processed for karyotyping prior to transplantation by a trained cytogeneticist. No abnormalities/aberrations were noted after *ex vivo* propagation. A representative ideogram is shown in the Supplementary data (Figure  3).

### 3.3. Clinical Assessment

Clinical assessment was performed on all patients based on 4 basic parameters of the UPDRS scoring system: (1) mental behavior and mood, (2) activities of daily living, (3) motor disabilities and impairment, and (4) complications of PD therapy. This was considered as the primary measurable outcome of the clinical study. The scoring was typically done during the “off” period (approx.12 hours off the anti-Parkinsonian medication) and during the “on” period (within 1-2 hrs of the medication) where maximum benefit could be appreciated in the PD symptoms. The average score during the “on period” at baseline was 62.33 and after stem cell transplantation it improved to 51.16 that is, an improvement of 17.92% over the baseline (Figure 2). Similarly for the “off period”, the average score was 86.5 at baseline and reduced to 59.5 after 12 months of stem cell transplantation. The percentage improvement in the “off period” score was 31.21% (Figure 3). This is similar to the data reported earlier by our group using autologous bone-marrow-derived mesenchymal stem cells [19].

Most of the PD patients reported subjective improvement during the first follow up that is, at 3 months after stem cell transplantation. These include clarity in speech, reduced tremors, and rigidity, and general sense of well being. These changes were seen in the later follow ups too indicating that the changes were not transient but more permanent. Similar improvements were also noted for some of PD plus patients but not all. However, for these patients the changes were transient and by the next follow up (6th month) most of them had progressed further into the disease.

### 3.4. Improvement in Relation to the Duration of the Disease

As depicted in Figure 5 and Table 2, there is a direct correlation observed between the duration of the disease and the improvements noted in the PD patients. Patients who had been diagnosed more recently performed better on the UPDRS compared to ones with the long-standing illness. Table 2 shows that those patients with PD for less than 5 years improved much quicker and remained in stable comparison to the patients suffering for more than 10–15 years. Whereas PD plus patients did not show improvement or such correlation after transplantation. Though there had been a subjective initial improvement, it never sustained in the long-term for PD plus syndromes.

It needs to be mentioned that the PD plus patients could not be rated using the UPDRS scoring system after stem cell transplantation due to the severity and progression of the disease. Hence, this data has not been mentioned.

**MRI Studies**

**Figure 4:**
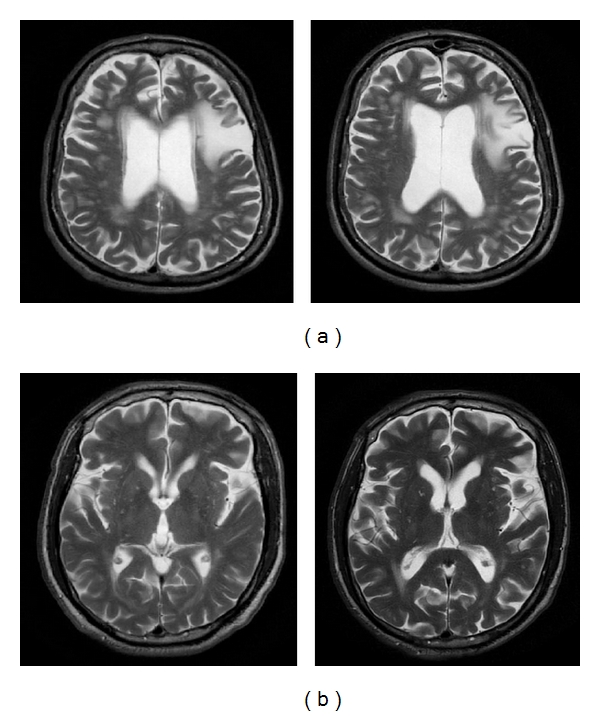
Shows T2 FLAIR axial images. (a) Depicts bilateral asymmetric multifocal hyperintensities involving pontine base periventricular and deep white matter suggestive of small-vessel ischemia. Diffuse brain atrophy is also seen with mineralization below globus pallidus and substantia nigra indicative of PD. There is no significant difference noted between the baseline and follow-up MRI. (b) Depicts moderate brain atrophy with bilateral putaminal rim sign seen. Small-vessel ischemic changes are seen in the bilateral periventricular and deep white matter. Mineralization of bilateral lentiform nuclei, dentate nuclei, and substantia nigra visualized is suggestive of MSA-P or PD plus syndrome. There was no significant difference noted between the baseline and follow-up MRI.

**Figure 5:**
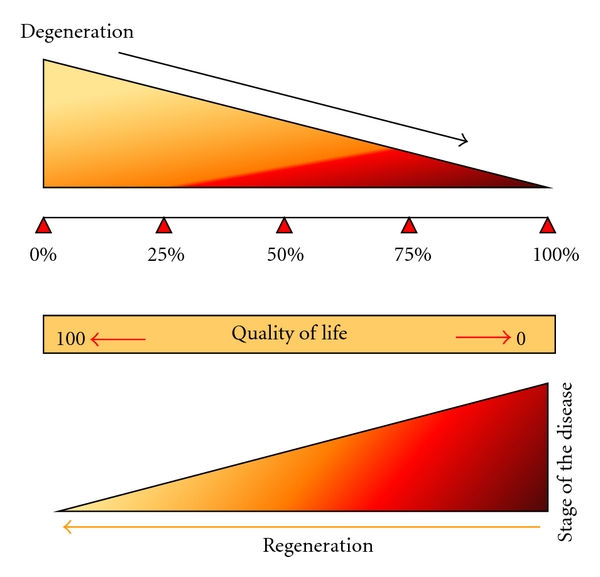
Depicts the balance between degeneration due to illness and the neuroregenerative reserve present in the brain. The schematic diagram illustrates that higher the degeneration higher the illness and more the regeneration required. Exogenous supply of stem cells midway between these two stages (i.e., 50%) would aid in improving the quality of life and reducing the disability due to disease.

MRI of the brain was done before and 12 months after stem cell therapy as shown in Figure 4. The brain images showed similar changes before and after treatment. No significant differences could be appreciated in the images. There were no structural changes, leukomalacia, or any additional growth observed. In one patient, incidental asymptomatic lacunar infarct was seen on follow up.

**MR Tractography**

The results of MR tractography have been shown in Tables 3(a) and 3(b). 12 different sites of the brain were analyzed during the different stages of follow up. A trend of improvement was observed in the genu and the peduncles steadily over a period of 12 months. The values improved from 0.53 ± 0.13 to 0.69 ± 0.07 in the right genu and 0.54 ± 0.14 to 0.63 ± 0.14 in the left genu and 0.50 ± 0.14 to 0.64 ± 0.04 in the left peduncle with a significant improvement on the 2nd follow up in the right peduncle. Interestingly in the PD plus patients, there was a further reduction in the values even after stem cell transplantation. It reduced from 0.378 ± 0.1255 to 0.3555 ± 0.1219 in the right genu and from 0.3875 ± 0.0723 to 0.3515 ± 0.1135 in the anterior limb of the internal capsule. 2 out of 4 patients have shown no improvement in FA values (i.e., FA values are decreasing in both the limbs of internal capsules on follow up scans). This correlated clinically with further deterioration of the symptoms in the PD plus syndromes.

### 3.5. Dose of Medication

The dosage of anti-Parkinsonism medication before and after stem cell transplantation was analyzed. For 4 PD plus patients, the clinician recommended an increase in dosage of medication based on their progression of the disease. However, for the PD patients in the early stages of the disease similar increase in dosage was not required. The dosage has remained the same as the baseline medications prescribed. This indicates that the disease has not progressed further after stem cell transplantation. Only for 2 patients, the dosage of Syndopa had to be increased. This is probably because the disease had already advanced beyond repair at the time of stem cell transplantation which is also evident from the UPDRS scores of the patients.

Therefore, out of the 8 PD patients, intervention was in the early stages in 6 patients. The progression of the disease appears to have been slowed after the administration of stem cells. They did not require enhancement of dose. In the late stages of disease and PD plus patients, stem cell transplantation had shown relatively lesser symptomatic relief and on the other hand needed an increase in medications.

### 3.6. Activities of Daily Living

Care givers have noticed an overall improvement in their activity levels in 7 patients, which includes reduction in tremors both at rest and in motion, better clarity in speech, reduction in rigidity, ability to walk for longer distances and perform personal tasks independently. This has a significant impact on the well being of the patient and further substantiates the fact that the progression of the disease has been slowed down after stem cell therapy.

## 4. Discussion

The current treatment for PD includes pharmacotherapies and deep brain stimulation techniques. Lesioning surgery is gradually fading. However, these can only produce symptomatic relief and have their own limitations and long-term side effects. Therefore, the need for alternative therapy is the need of the hour. Fetal nigral striatal grafts and neural stem cells are successful candidates used in the last few decades as a choice for PD. However, due to the fetal source it has ethical, immunological, tumorigenic risks besides sourcing concerns [8]. This has led scientists to explore further into the capacity of adult stem cells as a therapeutic target for PD since these are proven to be relatively safe, free of ethical issues, do not form tumours and have immunomodulatory potential. Studies have proved that both embryonic and adult stem cells *in vitro* can be transdifferentiated into functional dopamine secreting cells [14, 21, 22]. Animal data suggests that it is possible to transplant these cells into the brain and have therapeutic benefits in PD [23–25]. Hence, in this study we have chosen adult bone-marrow-derived mesenchymal stem cells.

In our first study [19], we have demonstrated the safety of autologous bone-marrow-derived mesenchymal stem cells transplanted unilaterally into the SVZ. However, with mixed results. Some patients showed improvement. There was an initial improvement period followed by a deterioration of the symptoms. This was possibly due to the continued degeneration in the nongrafted side. To nullify this effect, in the current study, we have undertaken bilateral stem cell transplantation.

During the earlier study we also noted that there is a difference in the population doubling time (PDT), morphology, differential potential, and cell senescence. Although the cells met the required standards of the ISCT, the PDT, and cell surface marker expression and differential potential was observed to be lesser than healthy donor BMMSCs. This may be attributed to the higher age of the patient where cells are known to have shorter telomere length [23] and lower proliferation potential. These cells also reached senescence *in vitro* much earlier (Passage 3) and hence it was challenging to be able to upscale the cells for transplantation (unpublished data). The mixed results obtained may be attributed to the differences in the cell properties observed.

In view of the preceding results, in this study we wanted to understand the safety and feasibility of bilateral “allogenic” bone-marrow-derived mesenchymal stem cells for PD. The rationale was to rule out bone marrow aspiration in the aging population of PD patients and the morbidity associated with it. In the current study, we have transplanted allogenic healthy donor mesenchymal cells at passage 2. These cells are easy to upscale *in vitro*, maintain differential potential and cell surface marker expression. It is believed that these cells will have potentially higher therapeutic benefits. Since these cells can be produced in a large scale, cell expansion would also help to make the therapy more affordable. And bilateral transplantation would prevent any further degeneration on the contralateral side.

After receiving appropriate approvals, the study was conducted in 8 PD patients and 4 PD plus patients. The small number was chosen to understand the safety of injecting allogenic adult bone marrow mesenchymal stem cells into the subventricular zone of PD patients.

### 4.1. Our Hypothesis

Parkinson's disease involves both the nigral and extranigral systems. As a result, there are motor complications, associated dementias, multiple system dysfunctioning, and decline in cognitive functions with time.

Most studies have focused on the motor aspects only which are due to the loss of dopaminergic neurons (DA) in the substantia nigra of the midbrain. Cell replacement experiments conducted till date are targeted towards the replacement of the DA neurons. The results of fetal mesencephalic transplantation show graft induced dyskinesias due to inflammation around the implants, mixed population of DA neurons, and inappropriate synaptic contacts [6, 8–10].

Therefore, in our study we opted to choose a cell type which would primarily help in neuroprotection of the affected region irrespective of the cell type, followed by neurogenesis. This is a more global approach to the problem which would target not only the classical motor symptoms but also associated memory loss and decline of cognitive functions. Our primary aim was to forestall the progress of the disease and secondly help in restoration of neural functions. This is the first paper to demonstrate that bilateral allogenic transplantation of adult bone-marrow-derived mesenchymal stem cells is safe and has beneficial neuroprotective and neurorestorative effects in PD patients.

In this context, it is imperative to understand how mesenchymal stem cells help in neuroprotection and neurogenesis.

It is well established that BMMSCs are capable of releasing cytotrophic mediators such as nerve growth factor (NGF) superfamily-Brain-derived neurotrophic factor (BDNF) and NGF-3; Glial-derived neurotrophic factor (GDNF) and neurturin [26]. These neurotrophic factors are essential for neurogenesis, neuroprotection, neuronal survival and differentiation. In PD animal models-mesenchymal stem cells are known to slow the progress of degeneration, improve neighbouring neuronal activity, regenerate nerve fibres, and most significantly induce proliferation and differentiation of the resident pool of neural stem cells [27].

Data from animal studies also demonstrate that inflammation at the SN of the midbrain leads to significant loss of dopaminergic neurons. Also there is a noted increase in the levels of tumour necrosis factors-*α*, interleukin 1*β* and *γ* interferon in the SN of PD patients [28]. Mesenchymal stem cells are known immunomodulatory. *In vitro* studies show that they are involved in immunosuppressive activities although the mechanism of action needs further clarity. In autoimmune encephalomyelitis animal models, mesenchymal stem cells have demonstrated a reduction in inflammatory infiltrates, lesser relapses, and neural insults. Recent studies are suggesting that NSAIDs are said to have beneficial effects in PD patients. An anti-inflammatory beneficial effect of mesenchymal stem cells is being proposed here.

The neural stem cells are located in the subventricular zone of the brain and hence is the most preferred site of injection in this study although invasive. The exogenous BMMSCs would help to activate and increase proliferation of the resident stem cells which has regenerative capacities (endogenous regeneration). At the same time, SVZ is far away from the known lesioning targets in the brain.

In this study, we report that there is 22% improvement in the UPDRS scores of the patients treated. The improvement was noticed only in the early diseased patients and not in PD plus. At the end of the study (12 months) it was not required to increase the medication which is an indicator that disease progression has been prevented. Further follow-up studies are on-going. No study till date has reported the ability to stall progression of PD in patients. Concurrent to the UPDRS scores, there was a sense of subjective well being perceived by the patient and caregivers in 10 out of 12 patients.

For PD plus, 3 patients have shown slight transient improvement post stem cell transplantation. In 1 patient there was no noticeable change. We feel that BM-mesenchymal stem cells should be considered as a treatment of choice in early-stage PD patients to appreciate maximum benefits. This is due to the loss of the “neuroregenerative reserve” present in the brain. It proves the fact that once the disease has progressed further and involves multiple areas, it is difficult to stop the process. Also the degeneration is so extensive and rapid that it is beyond the reparative capacity of the exogenous bone marrow mesenchymal stem cells to help in neuroprotection and neurogenesis. We also assume that the degeneration process is slow in the initial phases and gains momentum with time. PD plus appears to be involving multiple areas of degeneration *de novo* and hence none of the drugs or surgery are useful. Unfortunately cell therapy does not seem to alter the course. This creates a need for further studies where we need to consider the option of providing multiple doses of cells at frequent intervals and/or test the potential of stem cells derived from a different source like adipose tissue or umbilical cord matrix as these are also known to possess neuroprotective effects and higher transdifferentiation potentials. At baseline and 12 months after stem cell transplantation, MRI of the brain was performed. Although there has been improvement in symptoms and no further progression of the disease, there were no structural changes observed in the MRI scan. The MR tractography results show a specific pattern of recovery. Certain structural changes were observed in the genu of the corpus callosum and the left peduncle suggesting that early regeneration of the tracts probably occurs here. These changes are persistent throughout the follow-up study and clinically correlated with improvements reported in the patients. Currently we are continuing the study, with PET scans which will give us valuable information on any metabolic and functional changes happening in the SN region of the midbrain. This would give us further valuable clues in to the mechanism of regeneration in the human PD-affected brain.

Thus, to summarize, BM-mesenchymal stem cells have a three pronged therapeutic approaches in neurodegenerative diseases such as PD: neurogenesis, neuroprotection and neural plasticity. Also, it is essential to expose the degenerating brain to the exogenous stimulus of stem cells, while the *in situ* neuroregenerative reservoir of stem cells is present that is, in the early part of the disease.

## 5. Conclusion

This is the first paper to demonstrate that bilateral allogenic transplantation of adult bone-marrow-derived mesenchymal stem cells is safe and has beneficial neuroprotective and neurorestorative effects.

The study establishes the safety of adult bone-marrow-derived mesenchymal stem cell transplantation bilaterally into the subventricular zone of the human brain using burr hole surgery. There are improvements in the UPDRS scores of the PD patients, reported subjective well being and no increase in medications during the follow-up period. It is to be noted that no improvements were observed in the PD plus patients. This strengthens the fact that stem cell transplantation in the early stages of PD has the potential to prevent further progress of the disease. Results from this study suggest that allogenic BM-mesenchymal stem cells may be used as a disease modifying therapeutic strategy in treating PD. Unlike the known indications for surgical intervention, we recommend intervention in the early part of the disease to reap the best benefits. However, further long-term follow-up studies need to be carried out to understand the long term safety and sustainability of the benefit. Currently studies are going on to elucidate the mechanism of action of these cells in neuroprotection and neurogenesis in PD-affected human brain.

## Figures and Tables

**Table 1:** Shows the details of the PD patients and PD plus patients recruited for the study. It also provides the subjective improvements reported by the patient/caregivers after stem cell transplantation. It is to be noted that all patients have mentioned improvement in similar aspects of PD including reduction in tremors, stiffness of limbs and clearing of speech. However, for PD, plus there are no improvements noted.

Sr. no.	Age/ sex	Duration of disease (years)-PD/PD+	Date of SCT	Type of stem cells injected	Dose administered	ROA	Autologous/ allogenic	No. of injections	UPDRS score baseline off/on	12th month off/on	Comments by caregivers/patient
1	68/M	15-PD	Feb-10	BMMSCs	2 million	IC	Allogenic	1	154/127	128/127	NA

2	37/M	5-PD	Mar-10	BMMSCs	2 million	IC	Allogenic	1	79/47	52/50	(1) Following stem cell injection his rigidity had reduced and speech had improved

3	65/M	5-PD	Mar-10	BMMSCs	2 million	IC	Allogenic	1	94/58	28/22	(1) Able to do his routine activities without much difficulty (2) Stiffness and tremors are very minimal (3) Able to walk for longer distances

4	49/M	10-PD	Dec-10	BMMSCs	2 million	IC	Allogenic	1	112/98	Lost to follow up	(1) Patient is more independent in his daily activities (2) Speech is clearer (3) Rigidity and stiffness has reduced

5	54/F	10-PD	Feb-11	BMMSCs	4 million	IC	Allogenic	1	105/72	90/68	After therapy the patient and relatives have noticed that there was a decrease in involuntary movements

6	48/M	11-PD	Feb-11	BMMSCs	2 million	IC + IV	Allogenic	1	43/32	40/28	NA

7	56/M	5-PD	Mar-11	BMMSCs	2 million	IC	Allogenic	1	NA	NA	NA

8	60/M	5-PD	Feb-11	BMMSCs	2 million		Allogenic	1	44/38	19/12	(1) After therapy, he has notice d that the tremors have reduced (2) Walking difficulty has also reduced and (3) He is able to write better now

9	65/F	10-PD +	Mar-10	BMMSCs	3 million	IC	Allogenic	1	44/20	NA	(1) Speed of walking has increased (2) Slurred speech has improved (3) Swallowing is better (4) Slight Improvement in handwriting

10	66/M	7-PD+	Jul-10	BMMSCs	2 million	IC	Allogenic	1	62/62	NA	According to the family, the symptoms have worsened since a few months

11	69/M	3-PD+	Jan-11	BMMSCs	2 million	IC + IV	Allogenic	1	NA	NA	(1) 6 months after therapy, the patient and his relatives have noticed improvement in his rigidity and movements (2) There is also improvement in his speech

12	69/F	7-PD+	4-Nov-10	BMMSCs	2 million	IC	Allogenic	1	150/153	140/133	(1) At present her involuntary movements have reduced (2) She has difficulty in swallowing and stiffness in her neck has increased

**Table 2:** Shows the improvement in UPDRS scores for PD patients before and after stem cell transplantation.

Duration of PD	Percent of change in UPDRS scores	
(in yrs)	OFF period	ON period
5	34.17	6
5	70.21	62.06
5	56.81	68.42
10	14.28	5.55
11	6.9	12.5
15	16.12	1

**Table:** (a)

Site	FA values			
	Baseline	1st follow up	2nd follow up	3rd follow up
CSO-right	0.48 ± 0.09	0.46 ± 0.07	0.48 ± 0.15	0.50 ± 0.02
CSO-left	0.48 ± 0.07	0.48 ± 0.10	0.47 ± 0.11	0.51 ± 0.13
AL-right	0.42 ± 0.08	0.42 ± 0.09	0.36 ± 0.05	0.48 ± 0.19
AL-left	0.40 ± 0.07	0.36 ± 0.06	0.38 ± 0.08	0.33 ± 0.04
PL-right	0.66 ± 0.06	0.57 ± 0.14	0.64 ± 0.09	0.61 ± 0.16
PL-left	0.65 ± 0.11	0.60 ± 0.12	0.65 ± 0.07	0.61 ± 0.24
Genu-right	0.53 ± 0.13	0.56 ± 0.11	0.50 ± 0.16	0.69 ± 0.07
Genu-left	0.54 ± 0.14	0.58 ± 0.07	0.51 ± 0.11	0.63 ± 0.14
SPL-right	0.67 ± 0.12	0.69 ± 0.06	0.66 ± 0.11	0.65 ± 0.08
SPL-left	0.65 ± 0.19	0.73 ± 0.03	0.69 ± 0.10	0.59 ± 0.27
Peduncles-right	0.52 ± 0.13	0.53 ± 0.07	0.61 ± 0.08	0.56 ± 0.08
Peduncles-left	0.50 ± 0.14	0.56 ± 0.08	0.55 ± 0.05	0.64 ± 0.04

**Table:** (b)

Site	Baseline	Follow up
CSO-right	0.3995 ± 0.0881	0.395 ± 0.07125
CSO-left	0.43125 ± 0.398	0.44575 ± 0.08
AL-right	0.398 ± 0.0735	0.38475 ± 0.160
AL-left	0.3875 ± 0.0723	0.3515 ± 0.1135
PL-right	0.649 ± 0.0893	0.59475 ± 0.099
PL-left	0.66775 ± 0.079375	0.604 ± 0.10085
Genu-right	0.378 ± 0.1255	0.3555 ± 0.1219
Genu-left	0.433 ± 0.100	0.425 ± 0.1336
SPL-right	0.61 ± 0.1413	0.629 ± 0.118
SPL-left	0.63675 ± 0.14315	0.67 ± 0.13015
Peduncles-right	0.555 ± 0.126	0.60725 ± 0.149
Peduncles-left	0.512 ± 0.1016	0.53 ± 0.12065
